# Addressing diarrhea prevalence in the West African Middle Belt: social and geographic dimensions in a case study for Benin

**DOI:** 10.1186/1476-072X-7-17

**Published:** 2008-04-23

**Authors:** Saket Pande, Michiel A Keyzer, Aminou Arouna, Ben GJS Sonneveld

**Affiliations:** 1 Centre for World Food Studies (SOW-VU), VU University, Amsterdam, Netherlands; 2 Programme Analyse de la Politique Agricole (PAPA/INRAB), Porto-Novo, Benin

## Abstract

**Background:**

In West Africa, the Northern Sahelian zone and the coastal areas are densely populated but the Middle Belt in between is in general sparsely settled. Predictions of climate change foresee more frequent drought in the north and more frequent flooding in the coastal areas, while conditions in the Middle Belt will remain moderate. Consequently, the Middle Belt might become a major area for immigration but there may be constraining factors as well, particularly with respect to water availability. As a case study, the paper looks into the capacity of the Middle Belt zone of Benin, known as the Oueme River Basin (ORB), to reduce diarrhea prevalence. In Benin it links to the Millennium Development Goals on child mortality and environmental sustainability that are currently farthest from realization. However, diarrhea prevalence is only in part due to lack of availability of drinking water from a safe source. Social factors such as hygienic practices and poor sanitation are also at play. Furthermore, we consider these factors to possess the properties of a local public good that suffers from under provision and requires collective action, as individual actions to prevent illness are bound to fail as long as others free ride.

**Methods:**

Combining data from the Demographic Health Survey with various spatial data sets for Benin, we apply mixed effect logit regression to arrive at a spatially explicit assessment of geographical and social determinants of diarrhea prevalence. Starting from an analysis of these factors separately at national level, we identify relevant proxies at household level, estimate a function with geo-referenced independent variables and apply it to evaluate the costs and impacts of improving access to good water in the basin.

**Results:**

First, the study confirms the well established stylized fact on the causes of diarrhea that a household with access to clean water and with good hygienic practices will, irrespective of other conditions, not suffer diarrhea very often. Second, our endogeneity tests show that joint estimation performs better than an instrumental variable regression. Third, our model is stable with respect to its functional form, as competing specifications could not achieve better performance in overall likelihood or significance of parameters. Fourth, it finds that the richer and better educated segments of the population suffer much less from the disease and apparently can secure safe water for their households, irrespective of where they live. Fifth, regarding geographical causes, it indicates that diarrhea prevalence varies with groundwater availability and quality across Benin. Finally, our assessment of costs and benefits reveals that improving physical access to safe water is not expensive but can only marginally improve the overall health situation of the basin, unless the necessary complementary measures are taken in the social sphere.

**Conclusion:**

The ORB provides adequate water resources to accommodate future settlers but it lacks appropriate infrastructure to deliver safe water to households. Moreover, hygienic practices are often deficient. Therefore, a multifaceted approach is needed that acknowledges the public good aspects of health situation and consequently combines collective action with investments into water sources with improved management of public wells and further educational efforts to change hygienic practices.

## Background

**Figure 1:**
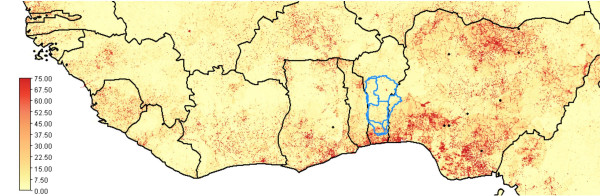
**Population density in West Africa.** The insert map shows infrastructure (black lines), the Oueme River (grey line) and settlements (dots) in the Beninese ORB.

In West Africa, the Northern Sahelian zone and the coastal areas are densely populated but the Middle Belt in between is in general sparsely settled (see Fig 1). The historical reasons for this phenomenon are only partly understood [1-3], and include explanations relating to slavery, to the high diversity and small size of tribes as well as to poor soil conditions. Nonetheless, the fact remains that the Middle Belt has underutilized land resources. Intensified settlement is already taking place at a significant scale from Northern regions that are threatened by encroaching deserts [4,5], and from the densely populated South where agricultural production capacity is endangered by nutrient mining [6-8]. Under climate change, this situation is most likely to worsen, with accelerated desertification in the North and more frequent occurrence of torrential rains and floods in the South, but much less in the Middle Belt proper. Consequently, the Middle Belt might become a major area for immigration.

Yet, several constraining factors need to be considered. Low population densities are often associated with poor institutional capacity, as low levels of investments into public services lead to poorly maintained water supply systems as well as to lack of hospitals, schools and sanitation facilities, and hence to various diseases, such as diarrhea. Each year it affects children in developing countries some 5 billion times, claiming the lives of nearly 1.8 million [9]. This annual death toll was in 2004 six times higher than from armed conflict on average in the 1990s and five times as many as from HIV/AIDS. Diarrhea also impedes weight gains in children, has adverse effects on their memory and their analytical skills and it reduces their school attendance, hence crippling their future [10].

Basically, the causes of diarrhea are well known and can be summarized as poor access to a good water source and poor sanitation. Besides threatening the life and future of children these causes also affect households in their budgets. One channel is through the cost of buying water, which often will be of better quality than from the available well, but unsafe nonetheless. This may take up to 12 percent of household income [9,10]. As is well known, getting safe water may also take much time of able-bodied members of the family and even at the expense of children's schooling time [10]. Existing inequities in access to good water and sanitation facilities can be expected to become more prominent as rising populations will depend on deficient and run down infrastructure.

In the presence of contagious diseases, particularly those that are water borne [11], health becomes a public good, as effective prevention and cure require collective action which should involve all people concerned. Hence households may not find it in their best interest to engage in prevention and cure when they expect to free ride on the efforts of others, and that others will do the same [12]. Consequently, public health in connection to these contagious diseases suffers from classical underprovision [13]. Furthermore, empirical studies such as [14] confirm that group interventions are needed and that closer connectedness of social networks under higher population densities is responsible for an increased transmission of diseases and results in higher diarrhea prevalence.

However, while the need for "participatory" and "community-based" approaches to prevent externalities from infections is now well recognized (e.g., [15]), the empirical evidence on the effectiveness of these community-based approaches is scanty [16]. In [17], the authors find that social interactions in driving latrine adoption by community members may have a significant and positive effect on a household's own adoption decision. In [18], a tendency for larger reductions in diarrhea prevalence is identified to occur at sites where the quality of the intervention was highest and had larger community involvement [19], suggest that marketing approaches, built on people's positive motivations may be more effective in promoting behavioral changes than traditional health education. It has been shown that these hygiene promotion programmes implemented at the community level are successful and that the changes in behaviour last longer [15]. Finally, mass communication campaigns for better personal hygiene have also improved hand washing participation [20]. In short, successful interventions to improve hygienic conditions cannot be done for target households separately and require a community-based approach as well as awareness campaigns [21].

Not surprisingly, the Human Development Report 2006 of UNDP assigns top priority to reducing diarrhea prevalence, improving access to good water sources and bettering the sanitary conditions. By now the donor community has started several "hand wash" programs aimed at increasing awareness towards good hygiene practices in developing countries, especially promoting hand washing with soap after defecation and before cooking [22,23].

Within West Africa, Benin is a particular case in point. By 2003, Benin counted a population of 7 million people growing at the very fast rate of almost 3 percent. At 51 years, life expectancy in Benin is one of the highest in West Africa. It had a GDP per capita (PPP) of 980 in that year with average annual growth (1990–2000) of 4.7 per cent for a population growth of around 2.7 percent. While Benin may be one of the poorest countries, its fraction of population below the 1 USD PPP poverty line of 37 percent is also one of the lowest in West Africa. Yet, the vast majority has poor sanitation facilities and only few households have asset ownership of refrigerators and electricity. Lastly, nearly 75 percent of the households have mothers that have no education [24]. In line with this, infant mortality rates are higher than the developed world: at 89.1 per 1000 and 77.8 per 1000 for children between 0–1 years and 1–4 years, respectively ([25], in [24]). Based on the DHS survey in 2001, diarrhea amongst children aged up to 5 years had a prevalence rate of 13.4 percent and 21 percent for children between 7–12 months [26]. High prevalence can also be partially blamed on co-morbidity with fever and pneumonia, possibly due to overlapping risk factors such as malaria endemicity [27]. Only 37.4 percent of households have piped water source and 43.2 percent obtain water from wells. Only 1.1 percent of the population has flush toilets, with 23.7 percent using pit latrines, while the rest use basic pit [24]. Hand wash programs were also started here [28], jointly with programs to increase the number and improve the management of water points and sanitation facilities [29].

However, physical geography matters also as some locations will be more vulnerable than others [30] and some currently safe sites are likely to lack adequate supply of water in the future. This further underlines the need for a spatial analysis of diarrhea prevalence and its contributing factors, which we conduct, for the reasons mentioned earlier, in the Oueme River Basin (ORB), the Middle Belt part of Benin that is likely to attract many more migrants in the future than it already does at present [31,32].

The common approach to study plausible causes of diarrhea prevalence is to relate the occurrence of the disease in a univariate analysis to climate anomalies [27], population density [24], household conditions [9], geo-spatial stress [33], whereas joint (i.e. multivariate) effects are rarely studied. Most studies either lack explanatory variables of geographic nature [34,35] or household specific variables [36], or both [30]. Many studies are location specific with findings that can not be extrapolated beyond the study area (e.g. [37,38,10]). Furthermore, policy makers are interested in a decision support system that facilitates identification of vulnerable regions so as to analyze the regional impacts on diarrhea prevalence and calculate the costs estimates of various interventions. Many studies focused on modeling in general lack such a provision.

Our paper intends to address abovementioned concerns of the diarrhea prevalence studies by combining data from the Demographic Health Survey [10,25] with various spatial data sets for Benin, and by applying mixed effect logit regression to arrive at a spatially explicit assessment of geographical and social determinants of diarrhea prevalence. After looking into these factors separately, at national level, we select from our household level database a set of explanatory variables that may serve as proxies for the social conditions and cross these with geographic circumstances. This enables us to estimate a function with georeferenced independent variables and apply it to evaluate, in a spatially explicit manner, the costs and impacts of better access to good water in the basin by increasing physical availability as well as by improvement in the social sphere.

The paper is structured as follows. Section 'Methods', presents a literature review on diarrhea prevalence and its possible causes. It discusses the data sources, including a comprehensive explanation on how the variables were constructed, the conceptual model, and the steps to implement it empirically through statistical procedures. Section 'Results' reports on the outcomes of the analysis. In particular, it discusses the estimation of the mixed-effect logit model that is applied to the ORB and comments on the policy interventions based on the model outcomes. The section 'Discussion' concludes.

## Methods

### Diarrhea prevalence: selection of variables

In the literature, diarrhea prevalence is attributed to factors of two kinds: household and geographical [39]. Among household variables, low mother's education is very widely identified as major factor to explain higher diarrhea occurrences in households [24,40,41]. Studies such as [42] established high correlation of diarrhea with household sanitation and hygiene conditions. Access of the household to good water has a positive effect in reducing diarrhea [9,10]. Distance to hospitals is also important, as access to medication is effective in controlling repeated occurrence of diarrhea [43]. Diarrhea occurrence is higher in lower income households where educational standards tend to be lower as well, access to good water source more limited, and hygiene practices poorer [44-46]. Many studies also emphasized the role of geographic variables such as climate [27], and especially rainfall [47], though the findings were not unambiguous. Studies such as [48,49] found higher infection rates during dry seasons, while others reported the same for the humid period [50,51]. For West Africa, the rotavirus infections, to which children below 5 years are most sensitive ([49] and references within), show a seasonal cycle that peaks mostly in drier months ([52] and references within), when water scarcity affects hygienic conditions and favors transmission via fecal-oral route [53].

The connection between accessibility to good water source and diarrhea prevalence is well documented [9]. For example, under ecological stress conditions, households tend to procure low quality water and cut down on water use for sanitary purposes [45]. Similarly, higher population densities coincide with intensified diarrhea prevalence, due to transmission of diseases via social networks that are obviously more connected when people are living closer together [14]. Households also often tend to substitute unreliable or inaccessible sources of good quality water by more reliable sources with poorer quality [54,55], the effect of which may even get more accentuated under higher population densities. Such tradeoffs appear when households dependent on groundwater find their aquifer unreliable or suffer from lower rainfalls on average.

Summing up, major factors explaining diarrhea prevalence would be household conditions such as income, mother's education, distance to hospital and sanitation conditions; and geographic conditions such as household's access to good water, population density, rainfall, ground water quality and suitability, and population density.

We report on how such variables were obtained for joint analysis in the present study.

### Data

#### Household data

The Demographic and Health Survey for 2001 constitutes one basic data set of the study [25]. It relies on multi-stage sampling design, stratified by region and urban/rural status, with sampling probability proportional to the population of selected enumeration areas (or clusters). DHS clusters are usually census enumeration areas, sometimes villages in rural areas or city blocks in urban areas. The coordinates of a cluster refer to the center of the corresponding settlement areas [56]. A total of 6219 women aged 15–49 years, sampled from 247 clusters were asked various question about health including those pertaining to children up to the age of 5 within their households. The question used to determine prevalence of diarrhea was whether a given child "Had diarrhea recently?" in the last 24 hours, last week, or longer periods. A total of 5796 households were covered by the survey. The DHS also comprises questions related to household specific characteristics, including the geographic cluster (enumeration area) to which the household belongs, as well as topics such as source of drinking water, existence of toilets, places of washing hands, disposal of rubbish, and condition of house. These information sources were used to create indicators of household characteristics and socio-economic conditions. Specifically, the household specific variables are constructed as follows.

##### Diarrhea prevalence (DA)

If response to the question "Had diarrhea recently?" is positive for "last 24 hours" or "last two weeks", then a value of 1 is assigned for that child. A value of 0 is assigned if response is positive for "No". Positive response to "don't know" is ignored and those entries are removed from the data set. If more than one child has an entry in the data set for the same household, an average value is taken.

##### Education of mother (ED)

If a mother in the household has any kind of education, ED = 1. For households with more than one mother, average of the indicator is used.

##### Hygiene condition of households (HG)

For the creation of a hygiene condition indicator, we assign a value of 0 for absence and 1 for the presence in a household of: 1. toilet facility 2. communal toilet facility 3. facility to wash hands 4. cleansing agents 5. basin and, 6. public/private removal of rubbish. An average of the assigned values is taken as an indicator of hygiene conditions.

##### House condition (HC)

As there is no direct indication of household's income in DHS, the condition of the house is used as indicator of its wellbeing. House condition is based on construction materials for floor, wall and roof. For example, if the house has a floor of natural material (earth, sand), a value of 0 is assigned, for wood planks, or cement a 1 is assigned. An average of the three values for floor, wall and roof is taken as an indicator of house condition.

##### Distance to the hospital (DH)

If there is any problem to reach a hospital, DH = 0. In case of multiple data entries for a single household, average value is taken.

##### Access to good water source (GW)

A household is having access to good water source when it has access to a public or a private well (GW = 1) and bad access to good water source otherwise (GW = 0). In our case households are taken to lack access to good water source when they depend on natural springs or streams, tankers, and rain water, which are generally of poor or unreliable quality.

##### Socio-economic indicator (Ind1)

A general indicator of household wellbeing is created from the above set of socio-economic variables by averaging ED, HG, HC, and DH for each household. Larger values of *Ind1 *indicate better household conditions.

Next, the values of these variables in the survey are classified into two classes to convert real valued variables into binary ones with unit value indicating favorable socio-economic condition. Specifically, household average values of DA, ED, HG, HC, and DH have thresholds of 0, 0.5, 0.3, 0.3 and 0. These thresholds are such that a non-negligible number of observations are available for each class. The total number of households with data available for all the variables is 2626.

#### Spatial data

The spatial data set that is used in this paper include maps of aquifer suitability, ground water quality, settlement population, mean annual rainfall, and county level diarrhea occurrence. The first two spatial data sets are obtained from [57]. They are created by spatial interpolation and classification of various groundwater attributes available in Benin-wide well data set. Population data is obtained for all documented and geo-referenced settlements in Benin [58]. Both these data sets are then used to create population-by-settlement maps as well as municipality-level population density maps, whereby a municipality (commune) refers to the second level of an administrative entity in Benin: a county (department), one administrative level higher, consists of several municipalities. Mean annual rainfall data set is obtained from [59]. County data on diarrhea occurrence was obtained from National Health Management Information System of Benin [60]. The co-ordinates of the centre of the geographic cluster are used to assign the relevant spatial data to a particular household.

The spatial variables are constructed as follows:

##### Access to good water source (GW)

Though GW is a household specific variable, it is used as a "quasi" geographic variable since it also represents the state of water management institutions when aggregated to cluster or higher administrative levels. However in the regression analysis, GW only enters at household level.

##### Aquifer Suitability Stress indicator (Geo1)

In order to construct this variable, we use GIS data of an aquifer suitability index created in [57], and district-level population density created from [58]. The aquifer suitability index in [57] is created by classifying interpolated values of hydraulic conductivity and type of aquifer into 4 classes from most suitable to least suitable. Most suitable aquifer is the one with unconsolidated rock type with high hydraulic conductivity while the worst suitable is the one with metamorphic rock type with lowest hydraulic conductivity. These indices are then further ranked into 5 equi-probable classes according to population densities of their respective locations, such that most suitable aquifer under lowest population density is most favorable geographic condition while least suitable aquifer under highest population density is least favorable. Finally averages over district constrained Thiessen polygons with cluster locations as centroids are taken so this index is becomes a cluster level variable.

##### Groundwater Quality Stress indicator (Geo2)

Similar to aquifer suitability stress indicator, we use GIS coverages of ground water quality indexes created in [57] along with district level population densities created from [58]. Groundwater quality index in [57] is created from interpolated ground water salinity levels and classified so that the lowest class corresponds to lowest observed salinity levels and locations with highest class correspond to highest salinity levels. These indices are further classified according to district level population densities in a fashion similar to the Aquifer suitability stress indicator to obtain groundwater quality stress indicator and, averaged over district-constrained Thiessen polygons with cluster locations as centroids.

##### Mean rainfall (Rf)

GIS coverage for mean annual rainfall is obtained from [59] and averaged over district-constrained Thiessen polygons with cluster locations as centroids. Thus, mean annual rainfall variable used in the analysis is cluster level.

All the spatial variables except GW considered here are at cluster level. Next, the two stress indicators and GW are consolidated via a multiplicative form into one variable that is used in regression analysis (see next sub-section 'Analysis'). This transformed variable is, therefore, at household level since GW is obtained at household level. In other words, for our analysis we take all households within the same cluster to face similar water stress and rainfall conditions but to differ possibly in their access to good water source. Households are differentiated further by socio-economic condition (ED, HG, DH). These variables are also consolidated into one socio-economic variable (called HH), the construction of which is explained in the next sub-section 'Analysis'. Finally, the dependent variable DA (diarrhea prevalence) is kept at household level as well.

### Analysis

#### Qualitative assessment

Before turning to regression analysis and simulation, we conduct a data visualization step to qualitatively assess diarrhea prevalence and its covariates. Several maps of diarrhea prevalence are created at different aggregation levels. Maps displaying cluster information are created from DHS survey both as estimates at cluster level based on the data for the households in these clusters and by municipality-constrained nearest neighbor interpolation of cluster level estimates. Next, variance decomposition is carried out to assess within and across cluster (or municipality) variance in diarrhea prevalence and its covariates, comprising both geographic and socio-economic conditions.

Furthermore, we conduct a qualitative analysis of the effects of various variables on diarrhea prevalence to gain insight as to which variables can best characterize the two geographic and socio-economic conditions and their effect on diarrhea prevalence, as in [51,61,37].

#### Regression Analysis and Simulation

The model specification proceeds as follows. We formulate a relationship that explains diarrhea prevalence on the basis of the conditions within households as well as their external, geographical environment.

We take households to be concerned with the health situation of their members but assume that they face difficulties in protecting them because contagion makes their actions ineffective unless the whole community acts collectively. Countering diarrhea through say, handwashing and cooking of water only helps if all comply who form part of the chain of communication of the disease. Against this background, the usual coordination failures on public (non-rival and non-excludable) goods, as caused by free riding and assurance problems, may, jointly with the rising population density, explain why in principle not very costly improvement in hygienic practices is so slow to materialize in actual practice.

Besides contamination, education also plays a role, and related to it the awareness of the dangers of unsafe water and the benefits from better hygiene. Though education is to a large extent a private good (as opposed to a non-rival one), it typically is offered jointly to the members of a community and remains fixed in the short term. Hence, it can be treated as an exogenous variable. Indeed, the policy emphasis on "participatory" and "community based" approaches has been emphasized in the literature referred to in the introduction precisely because of the intricate connections between individual and group level hygiene behaviour as well as the epidemiology of diseases itself.

Consequently, the household variables that may explain diarrhea prevalence should be interpreted as characterizing the average household in a collective of families with similar social conditions, as opposed to the actions individuals might take. In other words, they describe living conditions beyond the control of the individual and are, therefore, to be treated as exogenous.

To parametrize this relationship, we make use of a mixed-effect logit model, which is a generalization of standard logit model [62]. Probabilistic models have been used extensively in studies on disease prevalence, especially diarrhea ([61,63,64,34,46,36,35]) but as mentioned in the introduction few of these combine household with geographical information.

We also mention that joint consideration of factors at different spatial resolutions such as household specific variables (that are cluster varying) and geographic variables (that are cluster level) makes it possible to represent effects at local neighbourhoods as well as of interactions among these within the geographical units (as in variance analysis of [51]). This holds especially when households within a cluster may be positively correlated in diarrhea occurrence but have different living conditions as measured by the household characteristics considered [62,65]. In short, this mixed-effect logit model also accounts for unknown cluster specific effects in estimation.

We specify the mixed-effect logit as follows. Let each household *i *within cluster *j *contracts diarrhea, *y*_*ij *_= *1 *(else = 0), with probability *π*_*ij *_and events of diarrhea occurrence are postulated to follow a Logistic distribution. Probability of diarrhea occurrence, given cluster level and cluster varying factors *z*_*ij*_, is a Logistic function:

$$P(y_{ij}=1|z_{ij})=\pi_{ij}=\frac{e^{q_{ij}(z_{ij})}}{1+e^{q_{ij}(z_{ij})}},$$

for linear effect model

*q*_*ij*_(*z*_*ij*_) = *γ*^*T *^*z*_*ij *_+ *γ*_*o *_+ *ε*_*j*_,

where *ε*_*j *_~ *N*(*0*, *σ*^2^) and where, *z*_*ij *_is defined as a vector with elements,

*z *= {*z*^1^, *z*^2^, *z*^3^} = {[Geo_1 _(*2 *- GW)^2 ^Geo_2_], Rf,HH}

with,

Geo_1 _= aquifer suitability stress indicator,

Geo_2 _= ground water quality stress indicator,

GW = Access to ground water source

Rf = Mean annual rainfall in mm,

HH = a household indicator that sums the weighted binary household characteristics for HC (hygienic conditions), ED (mothers' education) and DH (Distance to Hospitals) whereby the sum of the weights, respectively, 0.25, 0.35 and 0.4, equals 1, obtained through minor calibration of each around value .33,

*γ *is a vector of parameters measuring the marginal effect of various factors in *z*,

*γ*_*o *_is a fixed intercept, and

*ε*_*j *_is normally distributed cluster-level random effect with 0 mean and *σ *standard deviation.

The dependent variable *y*_*ij *_assumes either a value of 0 or 1 and is defined by the, diarrhea prevalence (DA; see sub section 'Data'). The SAS procedure NLMIXED is used to implement the mixed effect model to estimate the coefficients *γ *and *γ*_*o*_, (see also for further discussion [66]).

We use this model for prediction at interpolated points on the settlement map using nearest neighbor estimates of independent variables. Based on the natural resource conditions and the cost and impacts of specific well drilling projects, we change the values of the covariates that relate to well accessibility, and compute the effect on diarrhea without changes in socio-economic indicators. However, before varying the level of accessibility specific independent variables through policy changes, we classify settlement population into three categories of equal probability (tertiles) based on household condition variables that also appear in the logit model. These are referred to as socio-economic classes in subsection on policy interventions.

## Results

### Preliminary maps

Figure 1 shows that population density distribution in the ORB is representative for the Middle Belt in West Africa: relatively low in the Northern part of the Basin and in higher in the South nearing the coastal areas.

**Figure 2:**
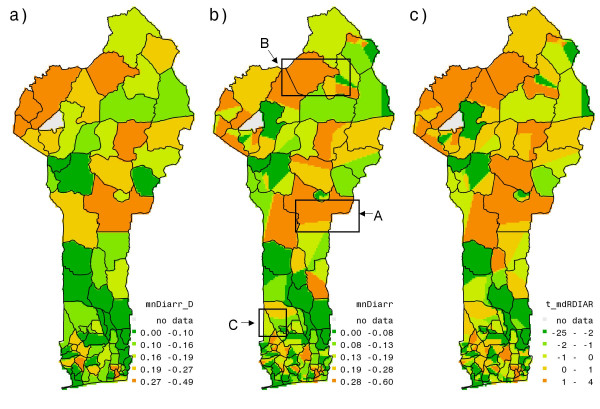
Diarrhea prevalence at (a) municipality, (b) diarrhea prevalence at cluster level, and (c) spatial difference in diarrhea prevalence across the country: deviation of diarrhea prevalence at cluster level from the national average.

Figure 2 has been created using Demographic and Health Survey data of USAID [25]. Data on diarrhea prevalence are obtained as the frequency in the survey [25] of households with a child that suffered diarrhea in the past 24 hours, computed within a cluster. The figure shows mean household prevalence of diarrhea averaged at the municipality level (Figure 2a) as well as cluster level (Figure 2b). From hereon, maps at cluster level refer to "municipality constrained" Thiessen polygon maps, which are created using nearest neighbor mapping from cluster locations to grids belonging to the same municipality. Here the prevalence rates reach even higher values, as much as 480 per 1000 children at municipality level. Overall, we observe almost the same spatial pattern in diarrhea prevalence as in Figure 1. The most affected municipalities lie in the mid-northwest and to a lesser extent in the south. Some municipalities in the south and mid-south, however, show lower diarrhea prevalence than in Figure 1. Yet, both figures concur on the parts of the country that face high prevalence of diarrhea. Figure 2c) makes this explicit by showing the differences of cluster level diarrhea rates from national average via t-statistics. The t-value for a cluster indicates whether this cluster value differs significantly from the national average, with a positive sign when cluster level value is higher than the national average. Hence, all three figures suggest some geographic pattern of diarrhea prevalence, with the middle and the northeast part of the country facing the least favorable conditions. However, since the survey data are collected at household level, there also is a social, in the sense of non-spatial component to be distinguished, that represents the variation across households at every location. Therefore, before considering possible explanations of these variations in terms of biophysical and "behavioral" factors, we decompose the diarrhea occurrence into a spatial and a non-spatial component.

### Variance Decomposition

The data set compiled from DHS is geo-referenced at cluster level. We estimate the part of total variance that is spatial by calculating the variance, across various clusters, of within clusters prevalence means, and similarly for clusters aggregated to municipality level.

Table 1 presents various descriptive statistics on prevalence of diarrhea in Benin, including this variance decomposition into *I*^*b *^the "variance between" and *I*^*w*^, the "variance within" municipalities. Both variances are significantly different from zero. Only 14 percent of the variance is associated with variance across clusters, whilst for municipality classes this percentage falls down to 8 percent, which could be expected as this enlarges the spatial class but it remains that even at cluster-level, the presumably non-geographic and non-biophysical "within-variation" is far superior to the not even exclusively geographic "between-variation". The next sections further develop this point.

**Table 1:** Key statistics on diarrhea prevalence in Benin. I^b ^is the "variance between" clusters or municipalities. I^w ^is the "variance within" clusters or municipalities.

Statistic		Value (t-statistics)	% of total
Mean		0.143	Not applicable
Variance		0.098	Not applicable
Cluster class	I^b^	0.015 (9.73)	14
	I^w^	0.091 (16.39)	86
Commune class	I^b^	0.008 (6.06)	8
	I^w^	0.092 (8.81)	92

### Spatial dimension of diarrhea prevalence

In this section, we show that despite the importance of within-variation, the between-variation that accounts for spatial variation cannot be discarded as this reflects quality differences among public institutions and cultural practices as well as of geographic conditions.

**Figure 3:**
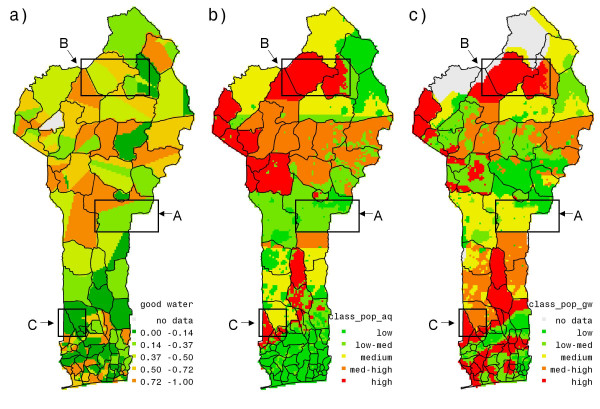
**(a) Accessibility to good water source, (b) aquifer suitability stress index, (c) ground water quality stress index.** Figure 3 (a) is created by averaging binary variable, GW, of accessibility to good water source at household level to the cluster level. In figures 3 (b) and (c) higher values indicate poorer aquifer suitability under higher population densities and poorer ground water quality under higher population densities respectively.

To represent the spatial variation of good access to water we averaged this binary variable at cluster level and depicted the results in Figure 3(a).

Figure 3 shows that many clusters in the northwest, mid-north and middle, and in the south of the country that have poor access to a good water source correspond to those with high diarrhea prevalence. However, not all the clusters with high diarrhea prevalence correspond to poor accessibility, as can be seen from boxes A, B, and C in the figures 2b and 3a. The same boxes, however, show in Figure 3(b) and 3(c) that high stress conditions prevail. Thus, households equipped with good source of water but unfavorable geographic conditions may face a similar situation as households with poor water accessibility. They tend to substitute their quantitatively unreliable sources with a less unreliable source, often of poorer quality. This increases their propensity to suffer from various water-borne diseases, such as diarrhea.

### Socio-economic dimension of diarrhea prevalence

Recall from Table 2 that only 14 percent of variance in diarrhea prevalence at cluster level is spatial while the remainder is "within-cluster" variance. We now look into factors such as mother's education (ED), hygiene condition (HG), income level (HC) and distance to hospitals (DH) that may help explaining this "within-cluster" variance in diarrhea prevalence.

**Table 2:** Summary statistics of the variables used for logit regression.

**Variable**	**N**	**Mean**	**Standard Deviation**	**Range (Min-Max)**
Diarrhea occurrence (DA)	2869	0.198	0.399	0–1
Access to good water (GW)	2943	0.636	0.481	0–1
Education (ED)	3017	0.271	0.445	0–1
Hygiene condition (HG)	3017	0.193	0.395	0–1
House condition (HC)	3017	0.793	0.405	0–1
Distance to hospital (DH)	2853	0.568	0.495	0–1
District mean annual rainfall (Rf)	-	1090	69.223	809–1225
District mean aquifer suitability stress (Geo_1_)	-	2.188	1.424	1–5
District groundwater quality stress (Geo2)	-	2.905	1.223	1–5

**Figure 4:**
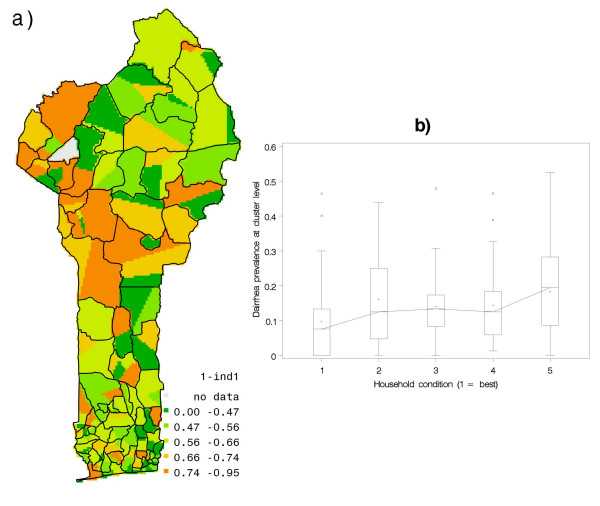
**(a) Indicator of household characteristics at cluster level, and (b) variability in diarrhea prevalence by socio-economic conditions at household level.** In Fig 4 (a), the indicator of household characteristics is closer to 1 for poorer household conditions. The x-axis in Fig 4 (b) shows 1-Ind1, classified into 5 equiprobable classes with increasing magnitude of 1-Ind1, wherein higher class values imply poorer household conditions.

Figure 4(a) shows [1 – Ind1] with higher values implying poorer household conditions, while figure 4(b) shows a box plot of [1 – Ind1] with increasing class number identifying poorer household conditions. This plot rejects any suggestion of diarrhea prevalence not being connected to household conditions. It also indicates that the variance in diarrhea occurrence across the classes remains the same but that the median of classes is non-decreasing (almost always) with poorer household conditions, and doubles from one extreme of household conditions to the other.

### Logit regression to model prevalence in the Oueme River Basin

This sub-section discusses application of the mixed effect logit model based on data for the entire country at household level, used to predict diarrhea prevalence in the Oueme River Basin (ORB).

The discussion so far has decomposed the prevalence of diarrhea into factors occurring at two levels, between and within clusters, with geographic or biophysical factors associated to the between-part, and socio-economic factors mainly but not exclusively confined to the within-part explanation. We now seek to go one step further, by estimating a logit model of diarrhea prevalence that includes socio-economic variables (such as DA, GW, ED, HG, DH) at household levels and geographic variables (geographic stress conditions Geo_1 _and Geo_2 _and rainfall, Rf) at cluster level, treating the household variables as exogenous as opposed to instrumentalizing them, for the reasons given earlier.

Regarding the linearity of the functional form of the mixed logit model (linear in three variables: geographic stress indicator scaled by accessibility conditions, rainfall and socio-economic indicator) we find that this specification is reasonably stable when nonlinear (square) terms are being allowed for. Likelihood estimates were found slightly lower with lower significance when covariates entered in square instead of linear forms. However, when covariates entered in both linear and square forms, corresponding parameters were found to be insignificantly different from 0.

Another linear functional specification ("Form2") with scaled geographic stress indicator as the only independent variable was also tested. While the resulting parameters were significant at 95% confidence level, the likelihood value was considerably lower than the linear form in variables functional specification ("original") with the latter additionally containing rainfall and socio-economic indicator as independent variables. This clearly suggests that additional variables explain additional variance in diarrhea prevalence. Furthermore, regarding our choice of explanatory variables, the likelihood improves as the variable "access to good water source" is supplemented by other covariates. This suggests that household variables influence diarrhea prevalence through other channels than accessibility and should therefore not be treated as instrument variables but rather as explicit explanatory variables. Next, we looked into the trade-off between joint estimation that improves the overall explanatory power as expressed by the likelihood value and possible bias on the treatment effect, as expressed by the Wu-Hausman test that might call for instrumentalization. For this, we conduct a two stage estimation with the first stage as a linear regression between the two covariates (rainfall and socio-economic indicator as independent variables) and geographic stress indicator scaled by "access to good water source" (as dependent variable); and use this regression function in the second stage mixed logit. First stage parameters are significant but the r-square (~0.072) is very poor, which, as reported by [67], tends to invalidate the use in IV-regression that is outperformed by OLS. The poor fit actually suggests that 'access to water' has ample room for variation at given level of the instruments, and can be used as an exogenous variable next to these instruments in the second stage regression, albeit that the Wu-Hausman test on first-stage residuals significantly deviates from zero, pointing to possible endogeneity.

In sum, given the low fit of the first stage estimation, the lower likelihood in the second stage estimation, the fact that no other variables of sufficient quality were obtainable from the DHS survey that could substitute as instruments and that the instruments have to appear in the structural equation anyhow, we conclude that joint estimation is the preferable approach to explain the diarrhea prevalence.

Table 3 shows the estimation results. Coefficients are all at least significant at 0.05 significance level and the hit ratio of correctly predicted classes is 76 per cent. All the fixed effects have expected signs. Improved household conditions reduce prevalence of diarrhea and its effect is significant. Geographic stress conditions accentuate prevalence of diarrhea, as households with similar socio-economic conditions and even similar access to good water source but less favorable geographic stress conditions have higher chance of facing diarrhea. Similarly, households with comparable socio-economic and geographic stress conditions have higher rates of diarrhea under circumstances of poorer access to good water source. Effect of rainfall on diarrhea prevalence is in line with other studies in West Africa as lower rainfall increases diarrhea prevalence.

**Table 3:** Parameter estimates *γ *of the logit model and overall model significance (log-likelihood ratio)

**Parameter Estimates**		
Variable	Estimate	t-score (Pr > |t|)

Geo-indicator^2^	0.006	1.98 (0.049)
Rainfall^3^	-0.004	-3.91 (<0.001)
Household-indicator^4^	-0.577	-2.94 (0.004)
Constant	2.786	2.52 (0.012)
-2 Log Likelihood	2447.6	
-2 Log Likelihood ratio^1^	325.4*	
Number of observations	2626	

^1^Under null hypothesis of a constant model, *significant at <0.0001 level. ^2^Product of aquifer suitability stress, groundwater quality stress, and (2-GW). ^3^Mean annual rainfall (mm). ^4^Weighted mean of ED, HG, and DH.

We conclude that the data show evidence of higher diarrhea transmission under scarcer water conditions in Benin, due to higher chance of human consumption of contaminated water.

Next, we apply the estimated model for prediction within the ORB. To compare the actual cluster-wise mean rate of diarrhea prevalence with the predicted one, we define the cluster-wise arithmetic mean of modeled prevalence: ${\bar{\pi }}_{j}=\frac{\sum_{ik,k=j}{\bar{\pi }}_{j}}{m}$, where ${\bar{\pi }}_{j}$ is the prevalence of diarrhea (*1 *or *0*) and *m *is the number of households in jth cluster.

**Figure 5:**
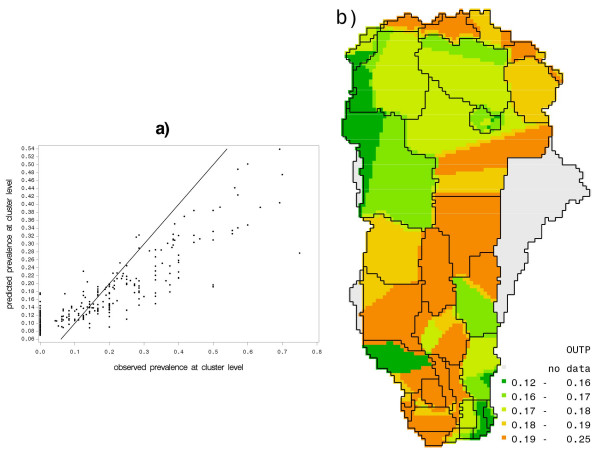
**(a) Scatterplot of observed versus predicted prevalence averages (at cluster level) for Benin, and (b) the predicted cluster-wide averages in the ORB.** The solid line in (a) is a 1:1 line, benchmark for a perfect fit.

Figure 5 compares the actual cluster-wise mean rate of diarrhea prevalence to the mean of the modeled one. Figure 5(a) shows the comparison of cluster-wise average of modeled prevalence within Benin and Figure 5(b) predicted cluster averages of prevalence rates in the ORB. We see in Figure 5a) that the model fits the observed average prevalence rates reasonably well with a correlation coefficient of 0.89. However, it appears to smoothen prevalence rates, under-predicting high levels while over-predicting the low ones.

**Figure 6:**
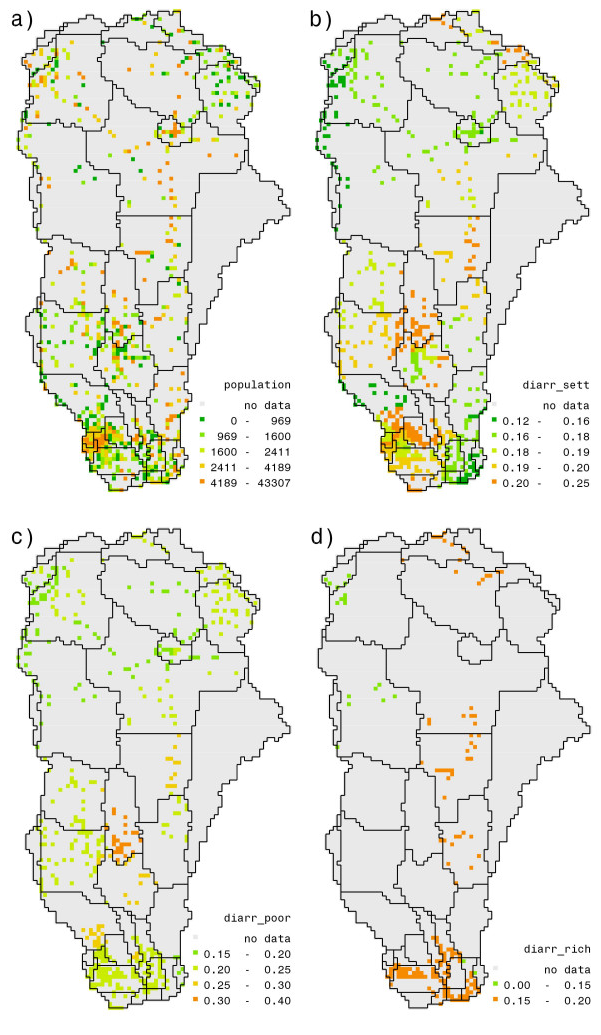
**(a) Population map of the ORB by settlements, (b) modelled prevalence map by settlements in the ORB, (c) average modeled prevalence rates for the lowest tertile, and (d) the highest tertile with poor access to good water source.** In figures 6 (c) and (d) prevalence rates are averaged over households of nearest cluster that have GW = 0.

The relationship between inter- and intra-cluster variables and diarrhea prevalence is induced from DHS survey data. However, as this data set contains information only for selected households that lie within a sample of cluster locations, we interpolate the relationship to all the settlements within the ORB by nearest-neighbor rule. Figure 6a) shows a population map by settlements and Figure 6b) a mean prevalence map. This settlement map will serve as reference for the remainder of the paper. The logit model clearly points to significant adverse effect of poor water access on prevalence of diarrhea, which depends among others on the stress conditions of the aquifers. However, the strength of this effect appears to depend on households' socio-economic conditions. In other words, the model confirms that at the same location the households with poorer socio-economic conditions have higher prevalence rates.

### Policy intervention: better access to good water for the poor

For the other explanatory variables in the logit model we use the values at the nearest cluster location, and given these values for explanatory variables evaluate the corresponding diarrhea prevalence. Figures 6c) and 6d) identify disparities in prevalence of two types. One is that while the highest as well as the lowest socio-economic tertiles (sub-section 'Analysis') face conditions of bad access to good water source, they suffer to a different degree.

Figure 6c) indicates that there are some households belonging to the lowest tertile in almost all settlements with poor water access and figure 6d) that very few settlements have their highest class with poor access. Besides this disparity in access, diarrhea rates are also higher for lower household class under conditions of poor access, suggesting once more that sanitation practices and education level of the households play a major role. This suggests that increase in accessibility conditions, irrespective of household class, reduces the probability of diarrhea occurrence but the effect of increased accessibility on diarrhea reduction is different for different household classes.

We have seen already that a policy intervention that improves accessibility reduces the probability of diarrhea occurrence. However, as is evident from figures 6c) and 6d), households belonging to the lowest household class are the most affected. To simulate the effects of such an intervention, we reduce the fraction of population within the lowest socio-economic tertile without access to good water from zero intervention levels to half of that, while keeping access frequencies of the other two household classes constant.

**Figure 7:**
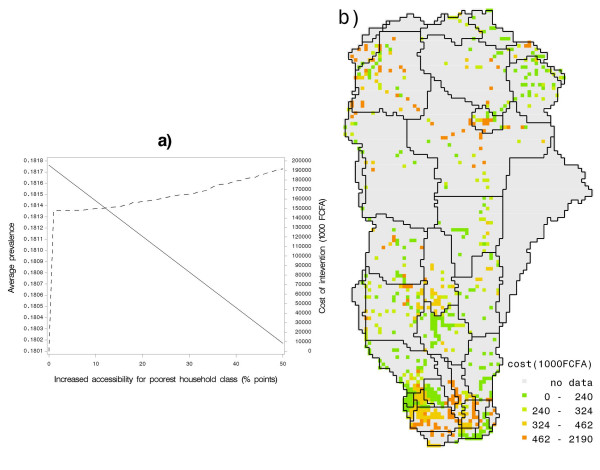
**(a) Change in average prevalence over the basin with policy intervention of increasing accessibility to the lowest household class (red) and total cost of these interventions (blue).** (b) Spatial distribution in the ORB for the cost of increasing accessibility of the lowest household class by reducing the fraction of households with poor access by half.

Figure 7(a) presents the result measured as prevalence of diarrhea for the lowest household class in the entire basin, against the cost of drilling wells, supposing that the increased accessibility is realized through addition of wells. Each such well is taken to serve at most 250 people in each of the settlements in the basin.

One remarkable outcome is the reduction of 0.17 percent only achieved at maximal intervention, from the average diarrhea prevalence in the ORB of approximately 18 percent. This leads us to conclude that, while necessary, drilling of wells that in principle can provide access to good water is far from sufficient to tackle the problem.

Figure 7(b) gives the associated spatial distribution of costs needed to deliver the stated improvement in access, depending on the number of people covered by the intervention, and location-specific information on aquifer properties [57]. The cost estimate for the construction of a well is based on drilling costs per meter depth as 5000 FCFA (1 EUR = 656 FCFA; 1 EUR ~1.6 USD) (community contributions; personal communication with experts from Direction de l'Hydraulique-Benin, [68] and [69]), multiplied by the drilling depth. For drilling depth, we use nation-wide groundwater inventories of [57] and selected from the maximum of water table depth or observed drilling depths. Finally, we consider a multiplicative factor that represents the aquifer suitability (rock type) conditions for each grid. Thus, total costs of drilling a single well, *C*_*s*_, at any grid location, *s*, can be formulated as,

*C*_*s *_= *5000*.*η*_*s*_(*q*_*s*_).*max*(*d*_*s*_, *w*_*s*_),

where for each grid s, *d*_*s*_: drilling depth, *w*_*s*_: depth to the water table, *η*_*s*_(*q*_*s*_): aquifer suitability multiplicative factor and *q*_*s*_: aquifer suitability.

The total cost for each location is based on the cost estimate of drilling a single well and the assumption that each well serves at most 250 persons. The figure shows that costs are highest in the south and some places in the mid-north as well as north-west. For the majority of the remaining basin, the costs of the intervention remain low.

## Discussion

The simulation exercise indicates that improvement in access to safe water on its own will not considerably reduce average diarrhea prevalence in the ORB. This may appear contrary to the meta-analysis of [10] who find that the overall pooled estimate indicated that water supply interventions are effective in reducing illness from diarrhea by 25 percent. However, our simulation was across space, as opposed to household level in the meta-analysis. More importantly, the meta-analysis was cross-sectional, including several countries and with only few villages per country. The study was conducted without instrumentalization, and, hence, can be expected to overrate the effect of interventions, since these are in a way treated like moving to another country altogether.

Our assessment of costs and benefits revealed that improving physical access to safe water is not expensive for the community as approximately 80 per cent of the cost is being subsidized by the government (personal communication Direction de l'Hydraulique, Benin). Community contributions are on average 11 USD per meter depth well drilling, which is relatively low compared to the total costs estimated on a 56–90 USD per meter [68,69]. Figure 7 also shows the prevailing favorable physical conditions for well drilling in the Basin. The fraction of lowest household class with poor access to water can be reduced by half at costs of less than 1000 USD per location (pixel) in 80 per cent of the locations, whereas on average the total costs of drilling a well varies from 1100 to 1800 USD in the rest of the country. Yet, as we learn from this study access to good water only marginally improves the overall health situation of the basin, unless the necessary complementary measures are taken in the social sphere. Consequently, to enhance the current living conditions and to improve the basin's capacity for absorption of future migrants to improve health conditions, initiatives to increase the number of water points with safe water [29] should go tandem with better management of wells and with educational programs such as "hand wash" program [28]. Indeed, policy makers should be provided with information from feasibility studies that analyze both access to good water (e.g. drilling wells) and social aspects (e.g. education, extension) as these interventions are only efficient in a joint implementation that can address the public good aspects of the issue. Possibly, the spatial variation of physical properties for well drilling in the ORB can also be used for an equitable distribution of the costs as community contributions at sites with favorable conditions can partially compensate for sites where drilling costs are high.

## Competing interests

The authors declare that they have no competing interests.

## Authors' contributions

SP, MAK, and BGJSS conceptualized the work. SP drafted the manuscript, and analyzed the data. SP, AA and BGJSS collected the data. AA and BJGSS contributed to manuscript drafting and critical review of the manuscript. MAK supervised the team on data collection, conceptualization and analysis and was the final editor of the manuscript. All the authors have read and approved the final manuscript.
